# Protection and immune modulation of activated human vaginal epithelial cells by *Aurea helianthus* extract

**DOI:** 10.1038/s41598-020-65821-4

**Published:** 2020-06-08

**Authors:** Yoonjin Park, Kyunghwa Lee, Chayul Lee, Ahran Song, Jinkwan Kim, Boyong Kim, SeungGwan Lee

**Affiliations:** 1 Department of Clinical Laboratory Sciences, College of Health Science, Korea University, Chuncheon-si, Republic of Korea; 2 Life Together,13, Gongdan-ro, Chuncheon-si, Gangwon-do Republic of Korea; 3 Mitosbio,13, Gongdan-ro, Chuncheon-si, Gangwon-do Republic of Korea; 4 Department of Biomedical Laboratory Science, College of Health Science, Jungwon University, Geo-San, Republic of Korea

**Keywords:** Biochemistry, Cell biology, Immunology, Plant sciences, Health care, Materials science

## Abstract

*Aurea helianthus* extract is associated with various properties including anti-melanogenesis, anti-oxidation, tumorigenic suppression, and immunoregulation; however, the mechanism by which it executes the immunomodulation of human vaginal epithelial cells (HVECs) remains elusive. We established three immunological functions of the extract. First, it mediated tumorigenic suppression in HVECs. Expression of cytokeratin 8, cancer antigen-125, and vimentin was dramatically downregulated in HVECs exposed to the extract under oxidative and fungal stresses. Second, the extract activated dendritic cells and macrophages. On exposing progenitor dendritic cells to the extract, the number of CD304^+^ cells increased by 40%; further, under oxidative and fungal stresses, this number was approximately 1.8 and 1.3 times lower, respectively, compared to that in the stressed cells. In monocytic differentiation, the number of dendritic cells and macrophages increased 9 and 6 times, respectively, compared to that in the control. Additionally, the extract enhanced and recovered polarisation by approximately 1.5 and 2 times, respectively, than that under stressed conditions. Third, the phagocytic activity of macrophages, against HPV16, 18, and 33 peptides, was enhanced by 12–35 times compared with that under stressed conditions. Thus, *A. helianthus* extract is a strong stimulator of the immune system and tumorigenic suppression under stress conditions.

## Introduction

Inflammation and cancer of the cervix and vagina are well known and highly incidental diseases worldwide^1^. These diseases are caused by various biological and non-biological factors. Notable causative agents include human papilloma virus, fungal protease, and free radicals such as hydrogen peroxide. Among the various types of HPVs, HPV16, 18, and 33 are high-risk types, and are responsible for 95% of cervical cancers^1,2^. Cytokeratins, known as intermediate filament proteins, are important cytoskeletal proteins. These proteins have well known mechanical functions and play various biochemical roles, such as controlling the cell cycle by transportation of p21 and regulating the expression of chemokine receptors, in several types of cells. Cytokeratin 8 (CK8) is a biomarker of cancer cells, and affects drug resistance and modulation of apoptotic resistance in cancers^3–5^. Vimentin, also an intermediate filament protein, is a cytoskeletal component of the mesenchymal cells and has been used as a biomarker for transition between epithelial and mesenchymal states in the development and metastasis of cancers. Vimentin plays various roles including maintenance of the cell shape, cytoplasmic integrity, cytoskeletal stability, and elimination of toxic proteins in mammalian cells. An upregulated expression of vimentin has been reported in various epithelial cancers including malignant melanoma, prostate cancer, breast cancer, and lung cancer^6,7^. The expression of cancer antigen-125 (CA-125), encoded by the *MUC16* gene, is upregulated in various cancers. This protein is located in the epithelial layer of the female reproductive tract and acts as a barrier against foreign particles and infectious agents in the tract. CA-125 is the most useful biomarker for detection of ovarian cancer. It is highly expressed in advanced ovarian cancers and has been used for screening^1,8,9^.

Dendritic cells (DCs) and macrophages play important roles in the immune system. DCs are essential for the modulation of initial T cell response and presentation of antigens to the immune cells^10^. The four categories of DCs include: conventional DCs (cDCs), Langerhans DCs (lDCs), plasmacytoid DCs (pDCs), and monocyte-derived DCs (mDCs)^10^. Macrophages have various functions including stimulation of naïve T cells, antigen presentation, phagocytosis, activation of neutrophils, tissue repair, and tumour inhibition^11–13^. M1 and M2 polarised macrophages encourage inflammation, anti-inflammation, and tissue repair. Macrophages are polarised through bidirectional interaction between macrophages and lymphoid cells. The M2 polarisation is driven by lymphoid cells, including T_H_2 cells and basophils, along with their cytokines, IL-4, IL-13, and IL-33^12,14^.

*Aurea helianthus* is a perennial herb belonging to the family Malvaceae, and its leaves, stems, flowers, and seeds are known to serve important functions^15,16^. All parts are edible, especially the leaves that have high iron, vitamin A, and vitamin C contents. The functions of *A. helianthus* extract include inhibition of tumour cells, anti-oxidation, modulation of lipid metabolism, anti-inflammation, anti-fever, immune regulation, detoxification, and anti-melanogenesis^15,16^. Although various functions have been reported, there has been no report on immune modulation of the human vaginal epithelial cells (HVECs) by the extract.

The goals of this study were to induce differentiation and activation of immune cells in the activated HVECs by *A. helianthus* extract, and tumorigenic suppression of the activated HVECs under oxidative and fungal stresses.

## Results

**Figure 1:**
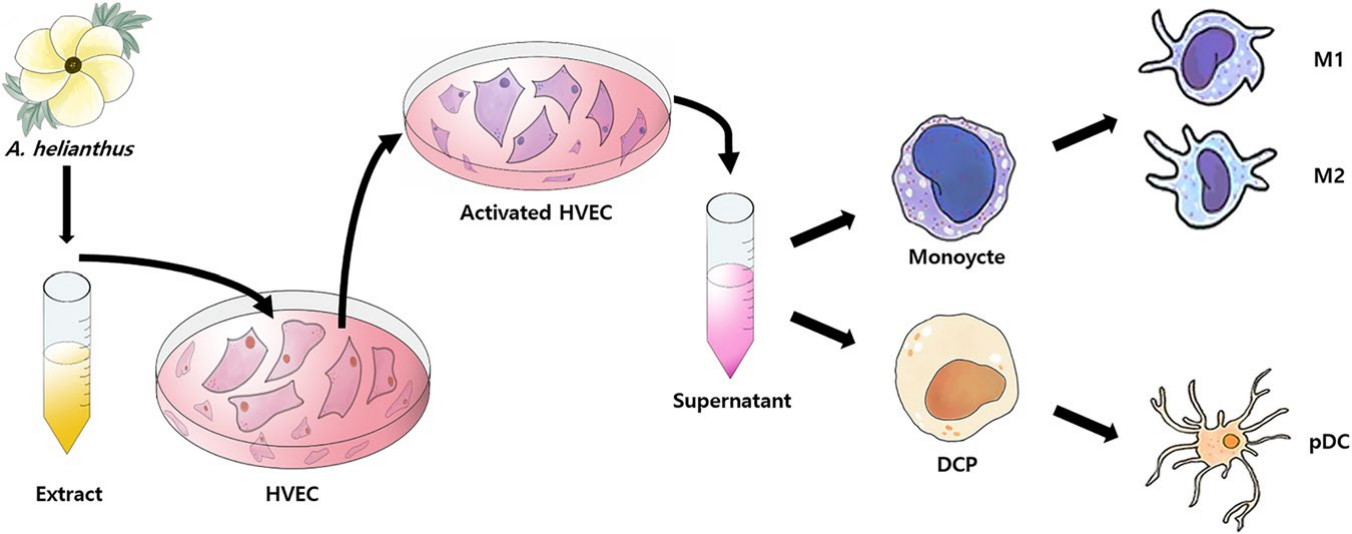
Schematic flow chart for the research procedures.Human vaginal epithelial cells (HVECs) were activated by the *Aurea helianthus* extract, and the supernatant of the activated cells was added to human monocytes and dendritic cell progenitors.

HEVCs, treated with the hydrolytic extract of *A. helianthus*, were activated to suppress the expression of tumorigenic markers, and the dendritic cell progenitors and monocytes were treated with the conditioned medium to enhance their differentiation (Fig. 1).

### Non-cytotoxicity and tumorigenic suppression of HVECs by A. helianthus extract

**Figure 2:**
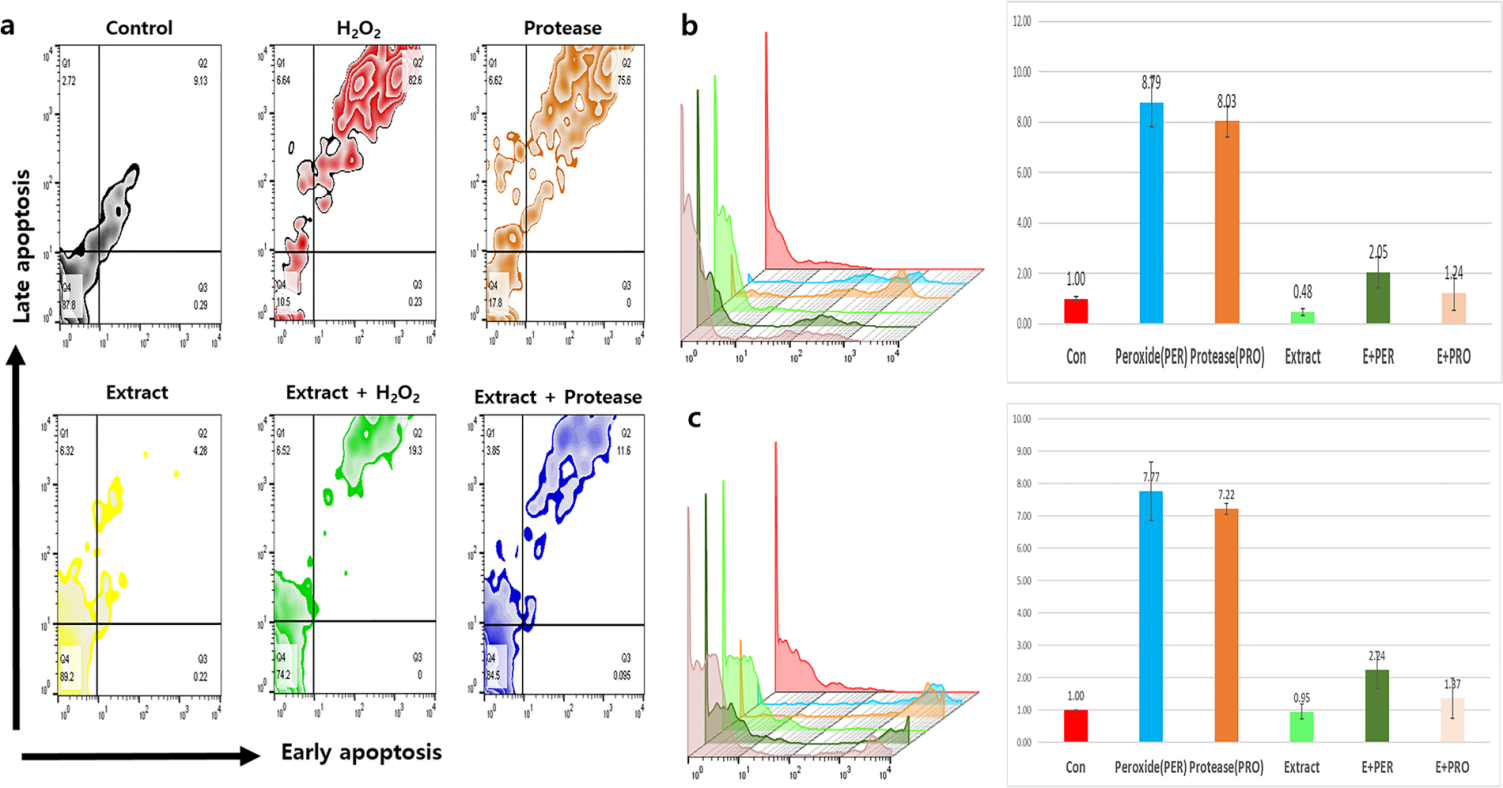
Anti-apoptotic effects of the extract in human vaginal cells under stresses. The *Aurea helianthus* extract-treated cells showed increased protective effects against hydrogen peroxide (50 µM/mL) and fungal protease (50 µg/mL). Panel (**a)** shows the results of flow cytometry, and panels (**b**) and (**c**) show the histograms and relative values for early and late apoptosis, respectively (*p* < 0.05).

**Figure 3:**
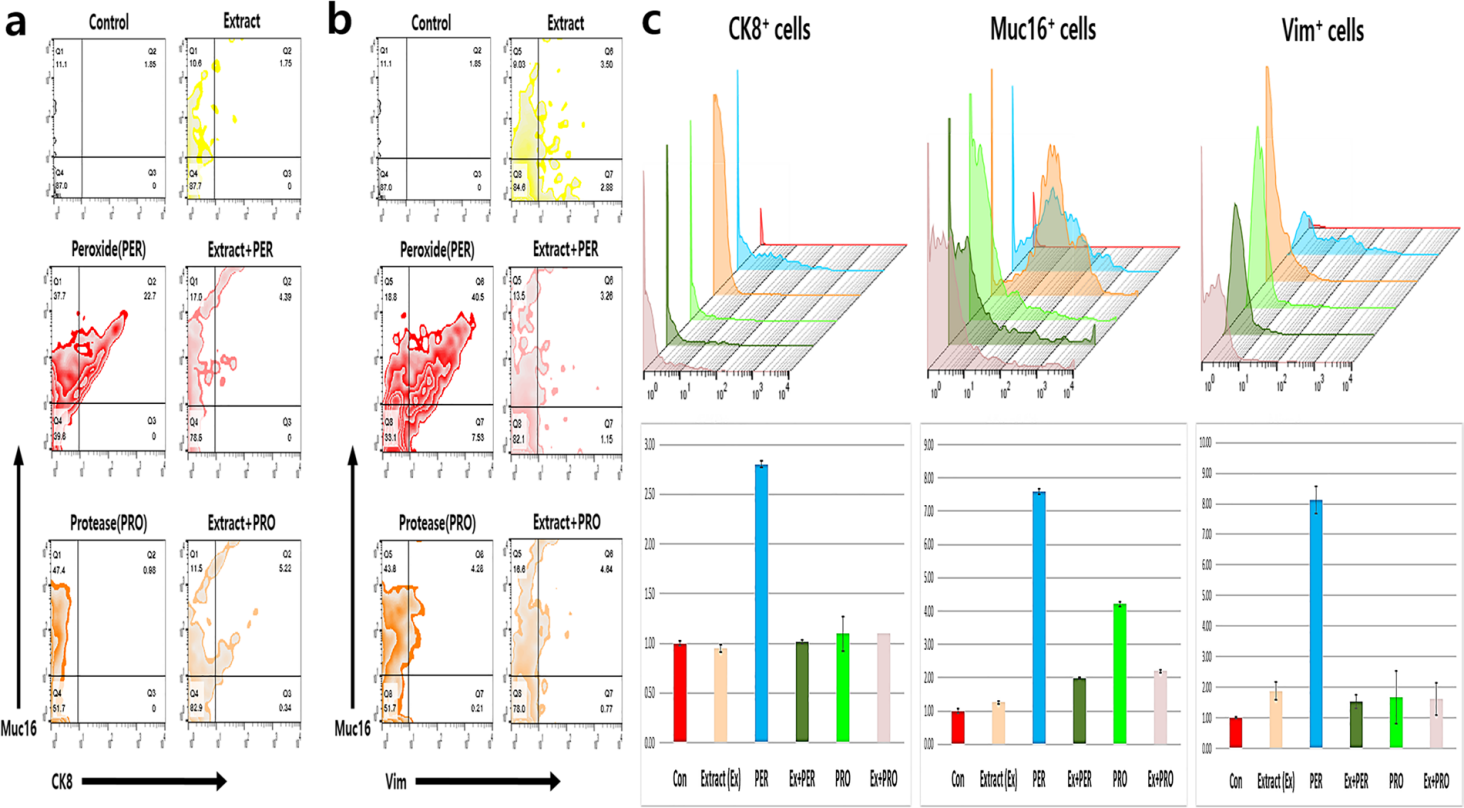
Protective effects of *Aurea helianthus* extract in human vaginal epithelial cells (HVECs) under stresses. The results indicate expression levels of the three stress markers in the HVECs under oxidative (H_2_O_2_) and fungal stress (protease). Panels (**a**) and (**b**) show the distribution of the three markers in the HVECs under the stressed conditions, and panel (**c**) shows the histograms based on the geometrical means of the three markers in the HVECs (*p* < 0.05).

Although HVEC proliferation in response to 800 μg/mL *A. helianthus* extract was similar to that in the control, under high doses of over 900 μg/mL, HVECs proliferation was 2.5 times higher than that in the control (supplementary data).The viability of HVECs, after treatment with *A. helianthus* extract (800 µg/mL), was found to be approximately 89.2%. The viability of non-treated HVECs, exposed to oxidative and fungal stresses, was approximately 10.5% and 17.8%, respectively. Interestingly, when the activated HVECs were exposed to these stresses, their viability increased by 7.1 and 4.8 folds, respectively, compared with that in the non-activated stressed samples (Fig. 2a,b). Moreover, compared to the non-treated cells, the expression of cancer markers including CK8, CA-125, and vimentin, was lower in the *A. helianthus* extract-treated cells. In the presence of hydrogen peroxide, the expression levels of the three markers in the activated HVECs decreased by 2.8, 3.8, and 5.5 folds, respectively, than those in the controls (Fig. 3a,b,c). In the presence of protease, a 2-fold decrease was noted in the expression levels of CA-125 compared with those in the controls (Fig. 3a,b,c). When exposed to the *A. helianthus* extract, the expression levels of Muc16^+^/CK8^+^ in HVEC population was dramatically lower than that in the stressed cells (Table 1). In the presence of hydrogen peroxide and protease, the expression levels of Muc16^+^/CK8^+^, Muc16^+^/Vim^+^, and Muc16^+^/CK8^-^ in the *A. helianthus* extract-treated cells decreased considerably (Table 1).

**Table 1:** Comparison of the expression levels of tumorigenic markers in human vaginal epithelial cells (HVECs) under various conditions.

Muc16* cells	Extract (Ext)	Peroxide (PER)	Ext + PER	Folds	Protease (Pro)	Ext + Pro	Folds
CK8^+^	1	12.97	2.51	5.17	0.56	2.98	0.19
CK8^−^	1	3.40	1.53	2.22	4.27	1.04	4.12
Vim^+^	1	13.28	1.08	12.35	1.40	1.59	0.88
Vim^−^	1	2.08	1.50	1.39	4.85	2.08	2.33

The values are shown as relative fold and are based on counting of target cells (%) by flow cytometry.

### Change in the differentiating pattern of dendritic cell progenitors by the conditioned medium

**Figure 4:**
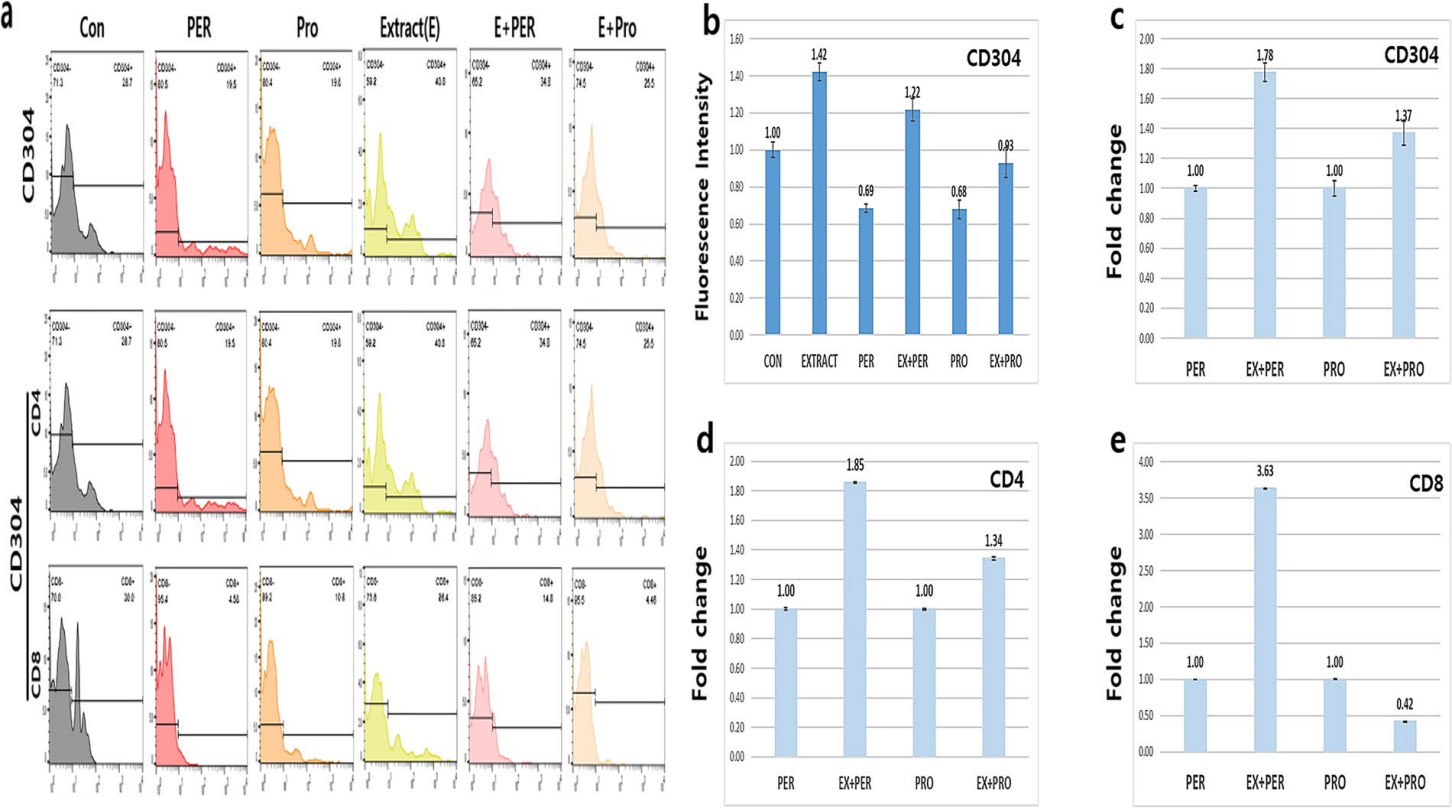
Differentiation patterns of progenitor dendritic cells by the activated vaginal cells. The dendritic cells were activated by the conditioned medium of the *Aurea helianthus* extract-treated human vaginal epithelial cells (HVECs). The histograms in panels **a** to **e** show the mean values of each samples for expression of the dendritic cell surface markers (*p* < 0.05). CON (control), Ext (the conditioned medium), PER (hydrogen peroxide), PRO (fungal protease).

The differentiation of dendritic cell progenitors was activated by the conditioned medium. Without the conditioned medium, the number of differentiated CD304^+^ cells was 30% lower than that in the controls under oxidative and fungal stresses; however, when exposed to the conditioned medium, the number of CD304^+^ cells was 40% more than that in the controls (Fig. 4a,b). Under oxidative and fungal stresses, the number of CD304^+^ cells was approximately 1.8 and 1.3 times lower, respectively, after exposure to the conditioned medium compared to that in the stressed cells (Fig. 4c). In addition to the CD304^+^ cells, the number of CD4^+^ and CD8^+^ cells was also approximately 1.8 and 3.2 times higher, respectively, compared to that in the oxidative stressed cells (Fig. 4d,e); however, only the number of CD4^+^ cells was 1.34 times higher than that in the fungal stressed cells (Fig. 4d,e).

### Change in the differentiating pattern and polarisation of monocytes by the conditioned medium

**Figure 5:**
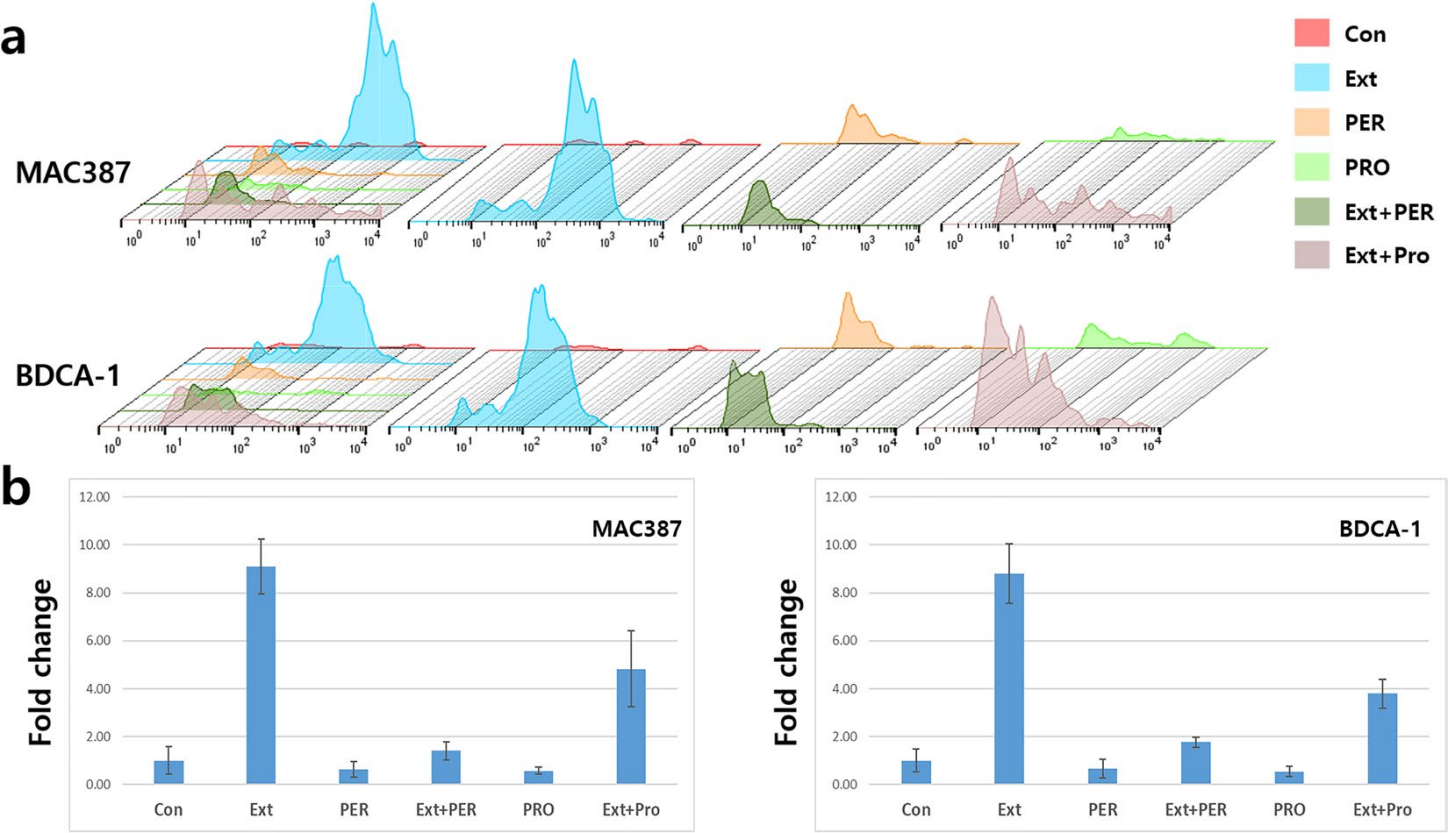
Monocytic differentiation by the activated vaginal cells. The differentiation of monocytes was activated the conditioned medium. The histograms in panel **a** show the geometric mean values of each sample for expression of the macrophage and dendritic cell markers. Panel **b** shows the fold changes in cell count of each sample. (*p* < 0.05). CON (control), Ext (the conditioned medium), PER (hydrogen peroxide), PRO (fungal protease).

The number of MAC387^+^ cells increased by approximately 9 times than that in the control in the presence of the conditioned medium, whereas the number was approximately 40% lower under oxidative and fungal stresses in the absence of conditioned medium (Fig. 5a,b). The differentiation of monocytes, exposed to the conditioned medium, was approximately 2.3 and 8.3 times higher under oxidative and fungal stresses, respectively, as compared to that in the stressed cells (Fig. 5b). Similar to the results for monocytic differentiation and DCs, the number of BDCA-1^+^ cells was approximately 8.8 times higher than that in the control in the presence of conditioned medium. Under oxidative and fungal stresses, the number of BDCA-1^+^ cells was approximately 40% lower than that in the control. Unlike the stressed cells, the monocytes exposed to the conditioned medium were actively differentiated to BDCA-1^+^ cells, and their number was 8.8 times higher than that in the control. Moreover, the number of BDCA-1^+^ cells was 1.8 and 3.8 times higher under oxidative and fungal stresses, respectively, compared to that in the control.

**Figure 6:**
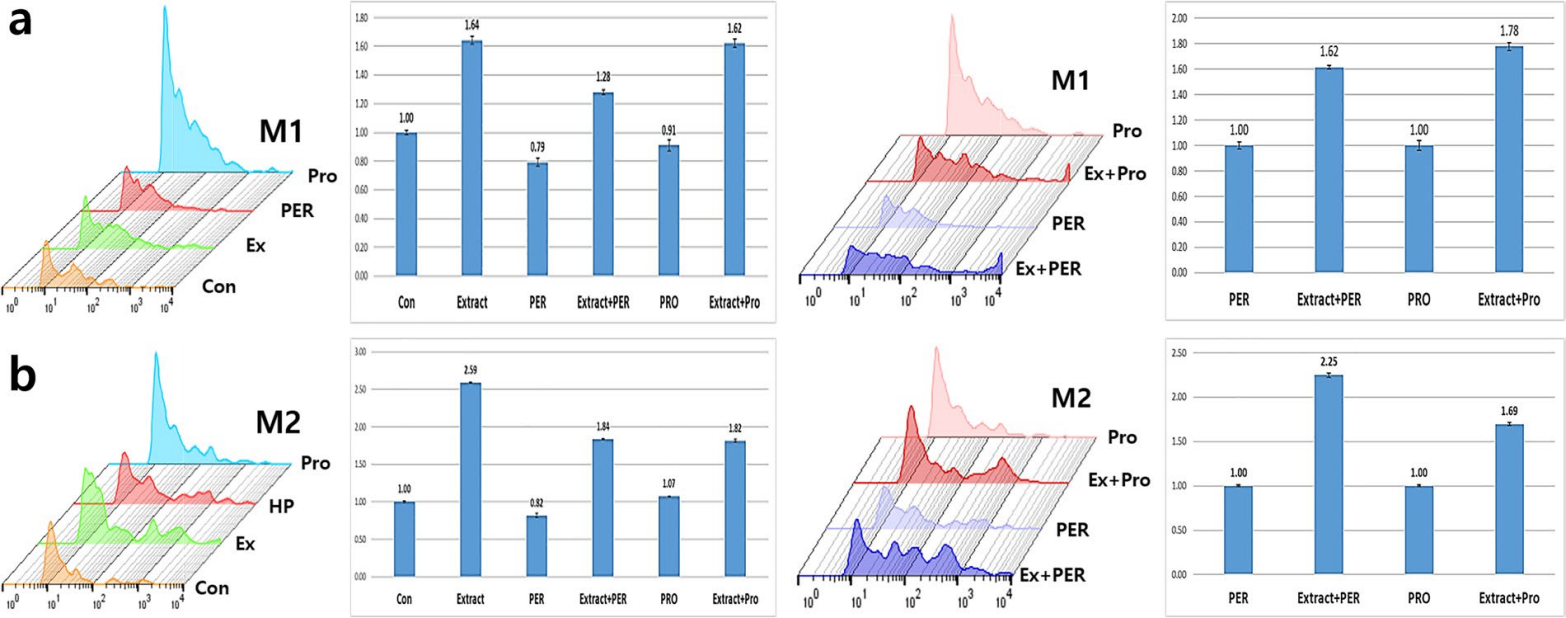
Polarisation of macrophages activated by the activated vaginal cells. The macrophages were activated by the conditioned medium under stressed conditions. The histograms in panels **a** and **b** show the geometrical mean values of each sample for expression of the markers, and the bar graphs show the relative fold change between the activated cells and the control and stressed samples (*p* < 0.05). CON (control), Ext (the conditioned medium), PER (hydrogen peroxide), PRO (fungal protease).

Additionally, the conditioned medium enhanced the polarisation of monocytes. Without the conditioned medium, the number of M1 macrophages was 20% and 10% lower than that in the control under oxidative and fungal stresses, respectively (Fig. 6a). The number of M2 macrophages was also 20% lower than that in the control under oxidative stress (Fig. 6b). The conditioned medium enhanced and recovered the polarisation by approximately 1.5 and 2 times under oxidative and fungal stresses, respectively (Fig. 6a,b).

### Activation of phagocytic activity by the conditioned medium

**Figure 7:**
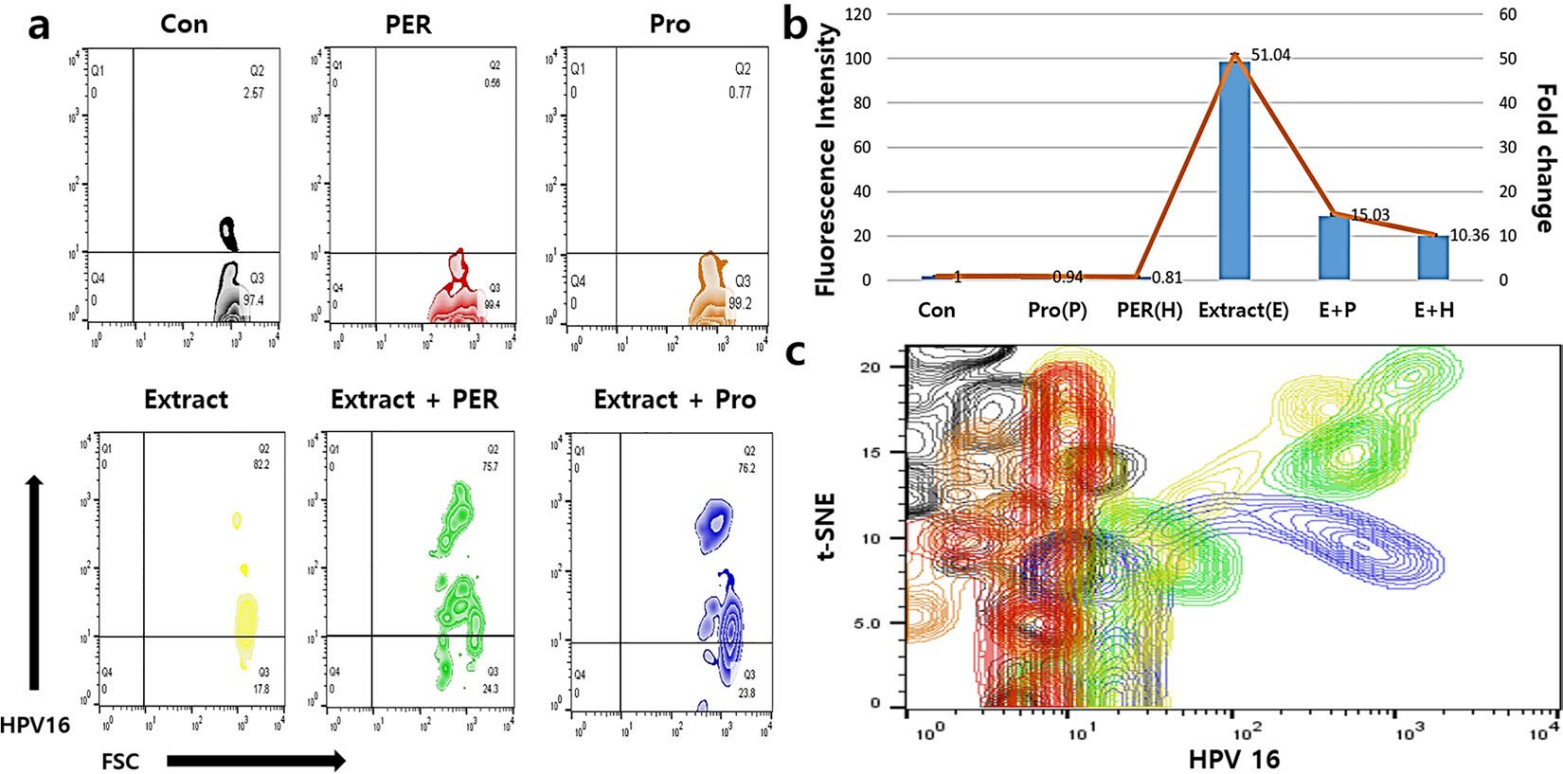
Phagocytic activity of the activated macrophages against HPV16 peptide. The macrophages were activated by the conditioned medium. The histograms in the panels show the mean fluorescence (**a**) and the fold values of each sample (**b**) for expression of the markers (*p* < 0.05). The t-SNE plot (**c**) shows the populations of HPV16 phagocytosed macrophages. CON (control), Ext (the conditioned medium), PER (hydrogen peroxide), PRO (fungal protease).

**Figure 8:**
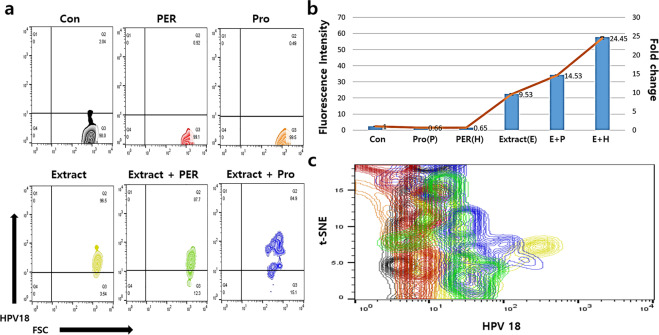
Phagocytic activity of the activated macrophages against HPV18 peptide. The macrophages were activated by the conditioned medium. The histograms in the panels show the mean fluorescence (**a**) and the fold values of each sample (**b**) for expression of the markers (*p* < 0.05). The t-SNE plot (**c**) shows the populations of HPV18 phagocytosed macrophages. CON (control), Extract (the conditioned medium), PER (hydrogen peroxide), PRO (fungal protease).

**Figure 9:**
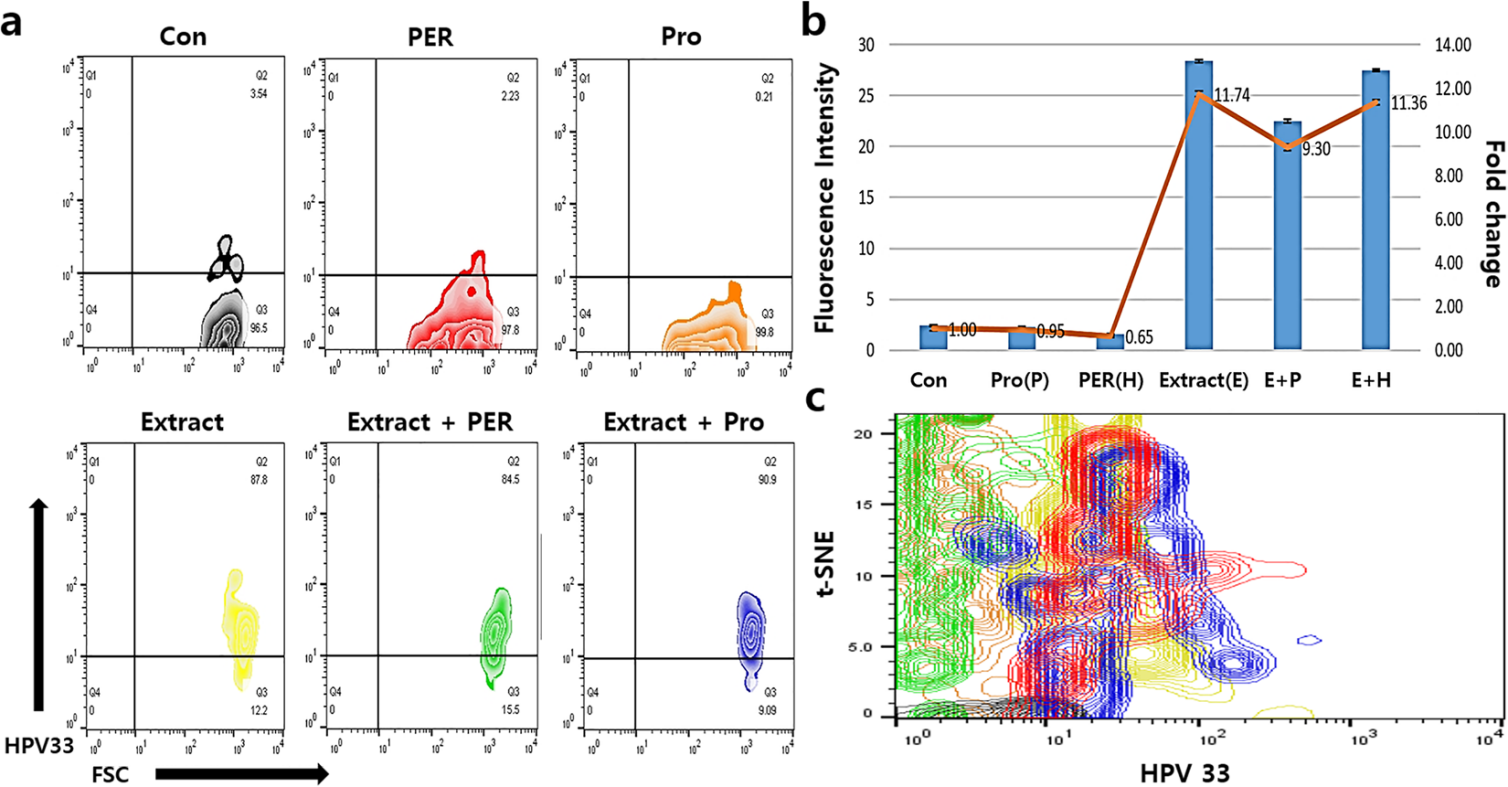
Phagocytic activity of the activated macrophages against HPV33 peptide. The macrophages were activated by the conditioned medium. The histograms in the panels show the mean fluorescence (**a**) and the fold values of each sample (**b**) for expression of the markers (*p* < 0.05). The t-SNE plot (**c**) shows the populations of HPV33 phagocytosed macrophages. CON (control), Ext (the conditioned medium), PER (hydrogen peroxide), PRO (fungal protease).

**Figure 10:**
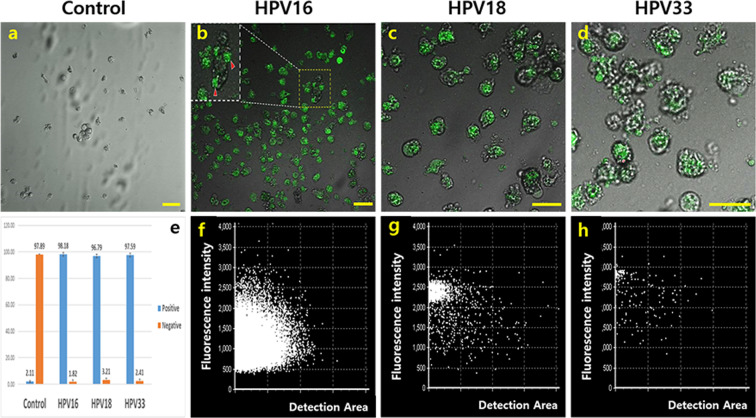
Enhancement of phagocytic activity of the macrophages against HPV peptides. The macrophages activated by the extract phagocytosed the FITC-conjugated HPV peptides (green). The HPV phagocytosed cells are stained green (**b**, **c**, **d**). The histogram shows the value of HPV phagocytosed macrophages (**e**). The **f** to **h** panels show the distribution of phagocytosed cells in the **b** to **d** panels, respectively (scale bar = 20 µm) (*p* < 0.05). The **a** panel is the control.

Surprisingly, the conditioned medium activated the phagocytic activity of macrophages. When exposed to the conditioned medium, the phagocytic activity of the macrophages against HPV16, 18, and 33, was 55 times higher than that in the negative controls, and the activity was 12–35 times more than that in the stressed conditions (the a and b panels of the Figs. 7, 8, 9 and Fig. 10). T-distributed stochastic neighbour embedding (t-SNE) revealed the populations with strong phagocytic activity to be present on the plots under the conditioned medium (Figs. 7c, 8c, 9c). In the presence of the conditioned medium, the phagocytic activity of macrophages was 24 times higher than that in the control, and phagocytosis was 30% and 15% lower under oxidative and fungal stresses, respectively (Table 2).

**Table 2:** Comparison of phagocytic activity of the activated macrophages against the HPV peptides.

HPV type	Extract (Ext)	Peroxide (PER)	Ext + PER	Fold	Protease (Pro)	Ext + Pro	Fold
HPV16	51.04	0.81	10.36	12.79	0.94	15.03	15.99
HPV18	9.53	0.65	24.45	37.62	0.66	14.53	22.02
HPV33	11.73	0.65	11.36	17.48	0.95	9.3	9.79

The values are shown as relative fold and are based on fluorescence intensity of macrophages measured by flow cytometry.

## Discussion

The global incidence of cervical and vaginal inflammation has been increasing continuously. Despite the availability of many medicines for treating these diseases, research aimed at identifying effective natural compounds or mixtures is crucial. According to the Chinese classical phytomedical book, Bencao Gangmu^17^, *A. helianthus* is an edible, well-known plant that can be used for the effective treatment of sexual organ diseases in humans. Studies on *A. helianthus* have established its functions such as anti-inflammation and anti-melanogenesis^15,16^. Additionally, these studies established that *A. helianthus* extracts activate and enhance the immunity of HVECs against carcinogens such as hydrogen peroxide and fungal protease. Under oxidative and fungal stresses, the viability of HVECs without exposure to the extract was 7 times lower than that in the control, and with exposure to the extract, the viability was similar to that of the control (Fig. 2). Even though the HVECs were exposed to the above stresses, the expression of cancer markers in HVECs was attenuated, owing to the extract exposure (Fig. 2). The concentrations of total phenols and flavonoids in the hydrolytic extract were slightly lower than those in organic solvent extracts (supplementary data). From some reports^18,19^ and supplementary data, high concentrations of vitamin E and phytoestrogen were contained in *A. helianthus*. These results suggest that the extracts contain phytomedical compounds such as vitamin E, phytoestrogen, phenols and flavonoids as shown in previous studies^15,16^ and that these compounds stimulate HVECs to enhance their protection against carcinogens as well as immunoactivity and cellular proliferation.

The conditioned medium also induced changes in the differentiation pattern of monocytes to DCs and macrophages, and the polarisation pattern of macrophages. Based on cellular surface markers, DCs are divided into four categories: cDCs, pDCs, lDCs, and mDCs^10^. Under inflammatory conditions, mDCs are activated to differentiate into several subsets and pDCs rapidly produce a large amount of type I interferon (IFNα or β) to defend against viral infection and further activate natural killer cells to secrete the interferon γ. pDCs also recruit other immune cells to the infected region^20–22^. With exposure to the aforementioned stresses, the differentiation of progenitor DCs was attenuated; however, the differentiation of the progenitor DCs, exposed to the conditioned medium, was enhanced even under the stressed conditions (Fig. 4). The conditioned medium activated the differentiation of progenitor DCs to pDCs (Fig. 4) and monocytic differentiation (Fig. 5) even under stress. Generally, pDCs form a bridge between innate and adaptive immunities^10^. Based on these functions, an increase in the number of pDCs means that the conditioned medium reinforces both the innate and adaptive immunities under stressed conditions.

Macrophage chemokines, CXCL10, induced by interferon gamma (IFN-γ), and CCL22, interacted with CCR4 protein, are secreted by M1 and M2 macrophages, respectively. Under bidirectional interactions between macrophages and T cells, CXCL10 from M1 binds to CXCR3 on the surface of T_H_1 cells, and CCL22 from M2 binds to CCR3 and CCR4 on the surface of T_H_2 cells. M1 macrophages enhance the antigen presenting capacity by killing the intracellular pathogens and causing tumour destruction. Unlike M1 macrophages, M2 macrophages modulate the immune system and activate clearance of parasites and tissue remodelling. When macrophages are stimulated by immunological factors including interferon γ, TNF-α, and interleukins 4, 10, and 13, they are polarised to M1 or M2 phenotype^13,14^. Upon exposure to the conditioned medium (Fig. 6), the polarisation of macrophages was encouraged and accelerated more towards the M2 phenotype than the M1 phenotype. When macrophages were exposed to the oxidative stress without the conditioned medium, the differentiation of macrophages to M1 and M2 phenotypes was attenuated by approximately 20%; however, under fungal stress, the attenuation of differentiation was only observed for the M1 phenotype (Fig. 6). These results (Fig. 6) suggest that activated HVECs secrete polarisation-activating cytokines such as interferon γ, TNF-α, and interleukins 4, 10, and 13. Moreover, the extract also contained functional compounds to induce secretion of cytokines from the HVECs. Besides enhancement of the polarisation, the conditioned medium-activated macrophages dramatically phagocytosed the HPV peptides (HPV16, 18, 33) even under stressed conditions (Figs. 7, 8, 9, 10). With exposure to the conditioned medium, the phagocytic activity increased 24 times of that in the control, and phagocytosis was 30% and 15% lower under oxidative and fungal stresses, respectively. E7 proteins, synthesized by cancer-associated HPV 16, 18, 33, bind to retinoblastoma tumour suppressor protein family members and accordingly prevent cell death, besides promoting cell cycle and replication of viral DNA. E7 proteins are also consistently expressed in human cervical carcinoma derived cell lines^23,24^. These results indicated that the phagocytic activity of the activated macrophages dramatically increased under stressed conditions (Figs. 7, 8, 9, 10). Further, *A. helianthus* extract has strong preventive effects on cellular immunity under oxidative and fungal stresses. Moreover, the results also suggest that *A. helianthus* extract performs anti-viral functions by enhancing phagocytic activity.

Consequently, *A. helianthus* extract has four important functions in HVECs under oxidative and fungal stresses. First, the extract is non-toxic for HVECs and suppresses the expression of tumorigenic markers in HVECs under stressed conditions. Second, the extract enhances the inflammatory immunity by inducing the differentiation of DCs to pDCs. Third, the extract activates the polarisation of macrophages, and fourth, the extract activates cellular immunity. Therefore, the extract is effective in suppression of cancer and metastasis, and activation of immune-modulating cells such as DCs and macrophages in the human vagina. In particular, these results suggest that *A. helianthus* extract can help to control virus-associated cancer risks. In further studies, we plan to study the functional compounds of exosomes from the conditioned medium.

## Materials and methods

### Cell cultures

Primary human vaginal epithelial cells (ATCC480-010, VA) were purchased and cultured in basal medium (ATCC 480-030) with a growth kit (ATCC 480-040). After fresh flowers having low temperature ripening at 10 °C for 48 h, the leaves of *A. helianthus* were dried with infrared ray for 2 h and grinded into microparticles (400 mesh). The powder was extracted with vacuum at 0.08 MPa, 70 °C for 2 h. After adjusting the treatment dosage of the *A. helianthus* water extract (800 µg/mL, cultured for 72 h), supported by Life Together (Gangwon-Do, Korea), the extract was added to the HVECs for 72 h at 37 °C, under 5% CO_2_. The supernatant of the treated cells, conditioned medium was collected. The activated HVECs were treated with hydrogen peroxide, 50 µM/mL (Daejung, Daejeon, Seoul, Korea), and fungal protease, 50 µg/mL (Sigma, MO, USA) for 2 h. The conditioned medium (100 µl/mL) was added to THP-1 cells (KCLB 40202, Seoul, Korea), which were cultured in RPMI 1640. Progenitor DCs (Lonza, MD, USA) were cultured with lymphocyte growth medium (CC-3211, Lonza), and after activation by conditioned medium for 24 h, the cells were exposed to the two stresses for 2 h.

### Cell apoptosis and viability

After exposure to the extract and stress factors, the HVECs were stained using the Annexin V-PI apoptosis detection kit (Invitrogen, MA, USA) to analyse cell viability using a flow cytometer (BD FACScalibur, BD science, CA, USA) and FlowJo 10.6.1 (BD science, CA, USA).

### Flow cytometry analysis

To analyse cell viability with a flow cytometer, all types of cells were fixed with 2% paraformaldehyde for 4 h^25^ and blocked with Fc blocking solution (BD science). Under the stresses, the activated HVECs were cultured for 1 day and treated with three immunoglobulins, FITC-anti-cytokeratin 8 (Abcam, Cambridge, UK), PE-anti-CA125 (Santacruz, CA, USA), and APC-anti-vimentin (Santacruz), after treatment with 0.02% Tween 20 for 5 min. The expression levels of the markers, in the treated cells, were analysed using a flow cytometer (BD FACScalibur) and FlowJo 10.6.1 (BD science). To analyse the differentiation of DCs, the progenitor DCs were fixed and treated with PerCP-anti-CD304, FITC-anti-CD4, and PE-CD8 (Biolegend, CA, USA) without the 0.02% Tween 20 treatment^26^. In the monocytic differentiation, FITC-anti-MAC387 (Novus, CO, USA) and APC-anti-BDCA-1 (Biolegend) were added to THP-1 cells without the 0.02% Tween 20 treatment.

### Polarisation of macrophage

To analyse macrophage polarisation, the activated monocytes, treated with 0.02% Tween 20, were exposed to the anti-mRNA probes, chemokines CXCL10 (Alexa Fluor 647-5′-GCTTCCAAGGATGGACCACA-3′) and CCL22 (Alexa Fluor 670-5′- GAGATCTGTGCCGATCCCAG-3′), and the stained cells were analysed using a flow cytometer (BD FACScalibur) and FlowJo 10.6.1.

### HPV phagocytic activity

After treatment with the conditioned medium, the monocytes were exposed to the synthesised FITC-conjugated HPV16 E7(83–97), LMGTLGIVCPICSQK; HPV18 E6(64–78), ACHKCIDFYSRIREL; and HPV33 E7(73–87), ASDLRTIQQLLMGTV (GL Biochem Ltd., Shanghai, China) peptides for 4 h at 37 °C and 5% CO_2_. After the incubation period, the phagocytic activity was analysed using a flow cytometer (BD calibur) and confocal microscope (FV-10, Olympus, Shinjuku, Japan).

### Statistical analysis

All experiments were performed thrice (n = 3), and the data were analysed by Independent T test, paired T test and analysis of variance (ANOVA), using the SPSS software v26 (IBM, NY, USA).

## Supplementary information

Supplementary Information.

## Data Availability

The datasets generated and/or analysed during the current study might be availed from the corresponding authors, on reasonable request.
