# Supplementary material for: Protection and immune modulation of activated human vaginal epithelial cells by Aurea helianthus extract
Source: Sci Rep. 2020 Jun 8;10:9227. doi: 10.1038/s41598-020-65821-4 (PMC7280265; doi:10.1038/s41598-020-65821-4)
Supplement: Supplementary file 1 — Supplementary Information. [file 41598_2020_65821_MOESM1_ESM.docx]

**Protection and immune modulation of activated human vaginal epithelial cells by *Aurea helianthus* extract**

Yoonjin Park^1^, Kyunghwa Lee^2,3^, Chayul Lee^2^, Ahran Song^1^, Jinkwan Kim^4^, Boyong Kim^1,2,3,4^*, SeungGwan Lee^1^*

^1^Department of Clinical Laboratory Sciences, College of Health Science, Korea University, Republic of Korea

^2^Life Together,**13, Gongdan-ro, Chuncheon-si, Gangwon-do**, Republic of Korea

^3^Mitosbio,**13, Gongdan-ro, Chuncheon-si, Gangwon-do**, Republic of Korea

^4^Department of Biomedical Laboratory Science, College of Health Science, Jungwon University, Geo-San, Republic of Korea

*Co-Corresponding authors; e-mail and phone: erythro74@korea.ac.kr, +82-10-9105-1435, seunggwan@korea.ac.kr, +82-10-9913-0147

Supplementary Data


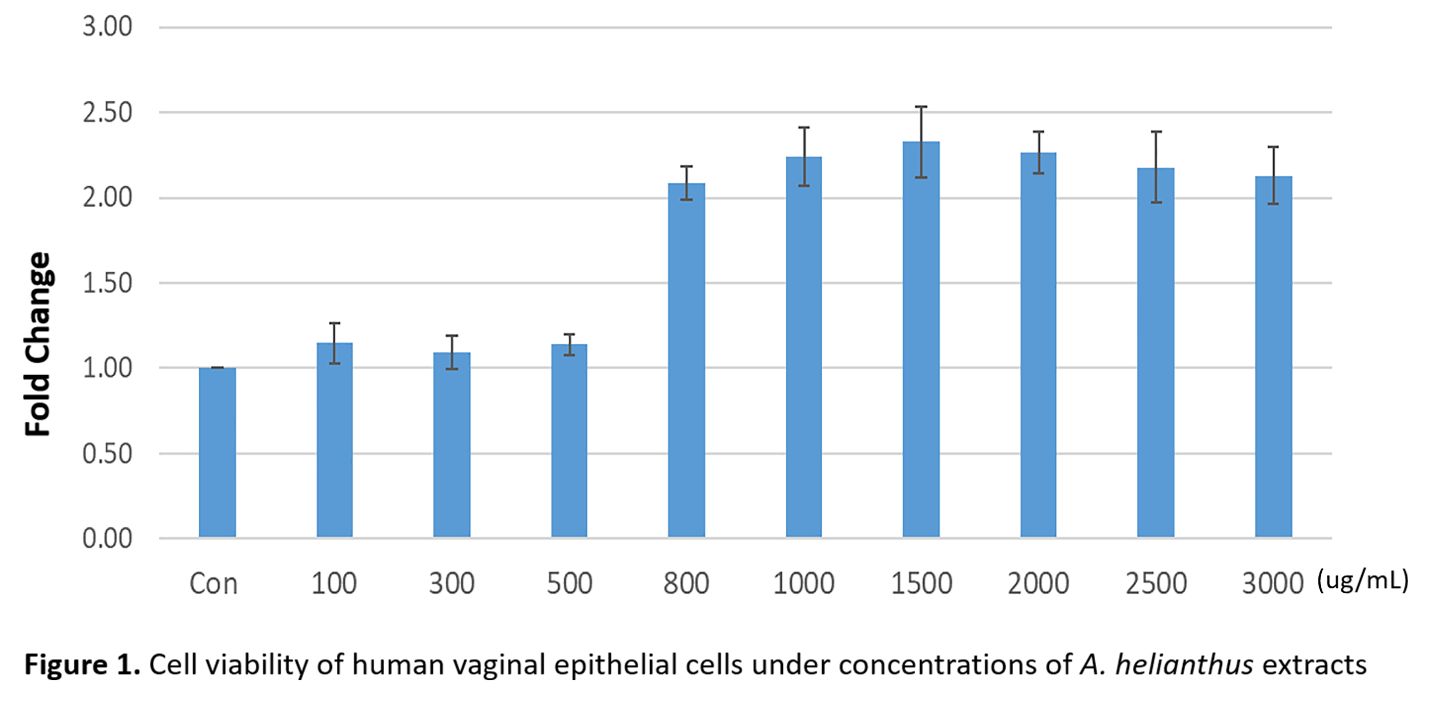


**Figure 1.** Cell viability of human vaginal epithelial cells under concentrations of *A. helianthus* extracts

HVECs exposure to over 800 ug/mL extracts of extract were proliferated about 2.2 times higher than the control for 3 days. This result suggest that hydrolytic *A. helianthus* extract is non-cytotoxicity and accelerated cell proliferation. Also, the concentration, 800ug/mL is the most optimal and effective concentration. (N=3, *P*<0.05)

**Table 1.** Measurements of total phenolic content and flavonoid content in hydrolytic and ethanolic extracts


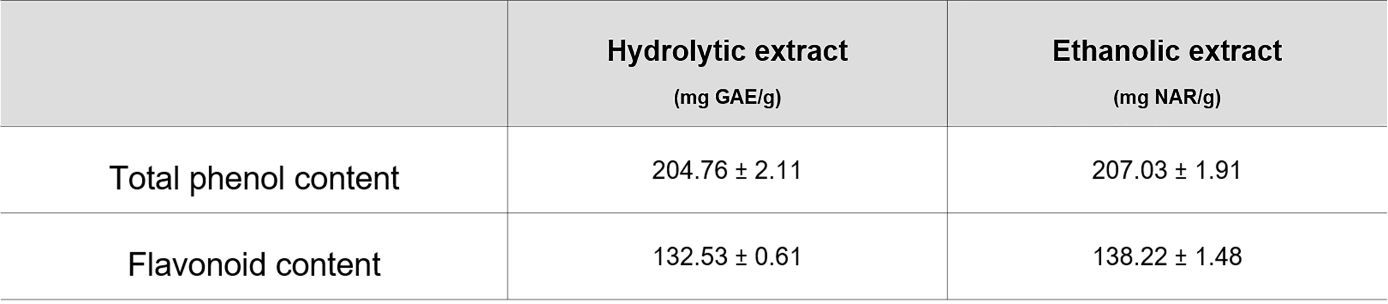


(N=3, *P*<0.05)

Unlike some reports (2019, [Hyon-il Ri](https://arxiv.org/search/q-bio?searchtype=author&query=Ri,%20H) et.al., 2017, Kim et.al) the concentrations of total phenol content (TPC) and flavonoid content in the hydrolytic extract were similar to the ethanolic extract.

**Table 2.** Measurements of quercetin, rutin and vitamin E in hydrolytic extracts of *A.Heliantus*


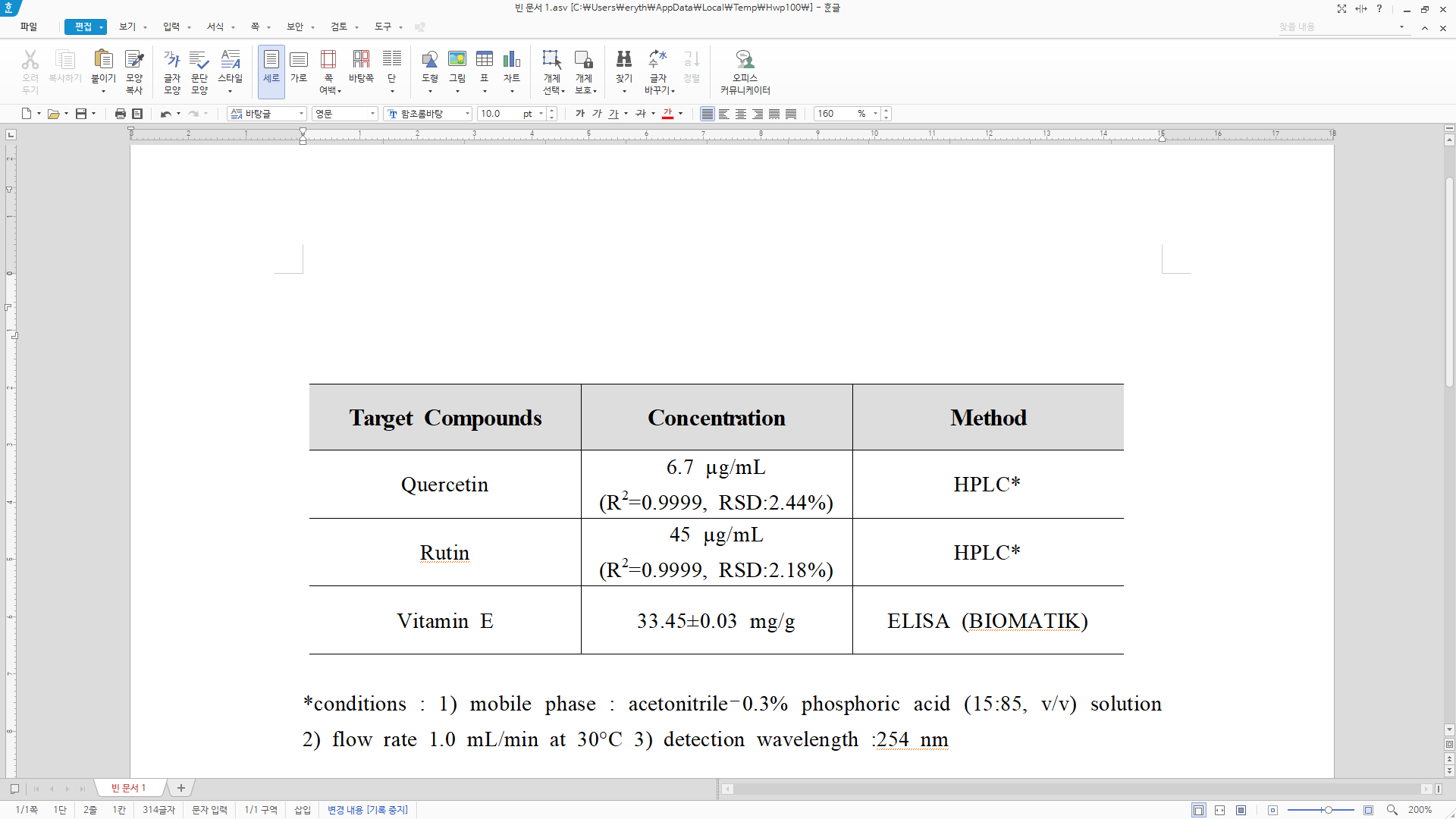


(N=3, *P*<0.05)

*Conditions: 1) mobile phase: acetonitrile–0.3% phosphoric acid (15:85, v/v) solution

2) flow rate 1.0 mL/min at 30°C

3) detection wavelength :254 nm

**References**

[Hyon-il Ri](https://arxiv.org/search/q-bio?searchtype=author&query=Ri%2C+H)., et al. (2019). “Purification of total flavonoids from Aurea Helianthus flowers and In Vitro Hypolipidemic Effect.” [arXiv:1906.12007](https://arxiv.org/abs/1906.12007)

Kim, H. J., et al. (2017). "In-vitro antioxidative, antiinflammatory properties of Aurea helianthus leaf extract a Korean traditional medicinal plant." Saudi J Biol Sci **24**(8): 1943-1947.
